# Melatonin Attenuates Contrast-Induced Nephropathy in Diabetic Rats: The Role of Interleukin-33 and Oxidative Stress

**DOI:** 10.1155/2016/9050828

**Published:** 2016-02-18

**Authors:** Didem Onk, Oruc Alper Onk, Kultigin Turkmen, Huseyin Serkan Erol, Tulin Akarsu Ayazoglu, Osman Nuri Keles, Mesut Halici, Ergun Topal

**Affiliations:** ^1^Department of Anesthesiology and Reanimation, Faculty of Medicine, Erzincan University, Erzincan, Turkey; ^2^Department of Cardiovascular Surgery, Faculty of Medicine, Erzincan University, Erzincan, Turkey; ^3^Division of Nephrology, Department of Internal Medicine, Meram School of Medicine, Necmettin Erbakan University, Meram Tıp Fakültesi No. 215, Meram, 42090 Konya, Turkey; ^4^Department of Biochemistry, Faculty of Veterinary, Ataturk University, Erzurum, Turkey; ^5^Department of Anesthesiology and Reanimation, Medeniyet University, Goztepe Training and Research Hospital, Istanbul, Turkey; ^6^Department of Histology and Embryology, Faculty of Medicine, Ataturk University, Erzurum, Turkey; ^7^Department of Cardiology, Faculty of Medicine, Erzincan University, Erzincan, Turkey

## Abstract

*Background.* Inflammation and oxidative stress (OxS) contribute to the pathogenesis of diabetic kidney disease (DKD) and contrast-induced nephropathy (CIN). Patients with DKD were found to be more prone to CIN. Interleukin-33 (IL-33) is a proinflammatory cytokine, but its role in DKD and CIN is unknown.* Methods.* Thirty male Sprague-Dawley rats were enrolled. The first group was comprised of healthy rats (HRs), whereas the other four groups were made up of diabetic rats (DRs), diabetic rats with contrast-induced nephropathy (CIN + DRs), melatonin-treated diabetic rats (MTDRs), and melatonin-treated CIN + DRs (MTCIN + DRs). All groups except the HRs received 50 mg/kg/day streptozotocin (STZ). CIN + DRs were constituted by administrating 1.5 mg/kg of intravenous radiocontrast dye on the 35th day. MTDRs and MTCIN + DRs were given 20 mg/kg/day of intraperitoneal injection of melatonin (MT) from the 28th day for the constitutive seven days.* Results.* We observed increased IL-33 in the kidney tissue following induction of CIN in DRs. To determine whether MT is effective in preventing CIN, we administered MT in CIN + DRs and demonstrated that kidney tissue levels of OxS markers, inflammatory cytokines, and IL-33 were significantly diminished in MTCIN + DRs compared with other groups without MT treatment (*p* < 0.05).* Conclusion.* Inhibition of IL-33 with MT provides therapeutic potential in DKD with CIN.

## 1. Introduction

Diabetes mellitus is the most common cause of chronic kidney disease worldwide. Diabetic kidney disease (DKD) can be seen in approximately 30 to 40% of both type 1 and type 2 diabetic patients [1]. According to American Diabetes Association (ADA) data, there have been 17.5 million diagnosed and 6.6 million undiagnosed diabetics in the United States [2], and this situation was found to be closely associated with both cardiovascular morbidity and mortality in this population [3].

Despite improvements in their chemical structure, contrast dye agents are still the third leading cause of hospital-acquired acute kidney injury. Reduction in the incidence of contrast-induced nephropathy (CIN) can lead to a decrease in morbidity, mortality (up to 36%), and length of hospital stay [4]. Diabetic patients are found to be more prone to CIN, which might further enhance the progression of DKD [5]. The exact pathophysiologic mechanisms of CIN are not fully understood; however, hypoxia and oxidative stress (OxS) constitute the main responsible pathways of this situation, especially in DKD [6]. Other important molecules that are closely associated with DKD and CIN are inflammatory cells such as leukocytes, monocytes, and macrophages-derived inflammatory cytokines, including interleukin- (IL-) 1*β*, IL-6, and IL-18 [7]. Recently, a new member of the IL-1 family, IL-33, is presented as an alarmin molecule that invites inflammatory cells to the kidney in cisplatin-induced nephropathy [8]. Accumulating evidence suggests that IL-33 is one of the most important cytokines in the pathogenesis of various disorders, including autoimmune diseases, myocardial infarction, heart failure, and allergic pulmonary diseases [9]; however, in the literature, the data regarding the role of IL-33 in DKD and CIN are scant. If a causative role of IL-33 is confirmed, preventing such inflammation and/or the immediate consequences of such inflammation may represent the key target to prevent DKD.

Various treatment modalities have been introduced to overcome DKD and CIN. Although melatonin (MT) is one of the attempted molecules in DKD, the role of this drug in diabetic patients with CIN remains unclear. Hence, we sought to determine the levels of IL-33 along with other inflammatory cytokines and OxS. The second aim of the present study was to determine whether MT has a preventive effect on kidneys in diabetic rats (DRs) with CIN treatment.

## 2. Materials and Methods

The study protocol was approved by the Medical Ethics Committee of Erzincan University (Mengucek Gazi Training and Research Hospital, Erzincan, Turkey).

Thirty male Sprague-Dawley rats were obtained from Ataturk University Medical Experimental Application and Research Center. The rats were housed in a standard environment with a room temperature of 22 ± 2°C, humidity level of 40–60%, and 12/12 h of light-dark cycle. Before starting the experimental procedure, the rats were acclimated to our facilities for one week. After completion of the acclimation period, the rats were randomly allocated into five groups. The first group was comprised of healthy rats (HRs), whereas the other four groups were made up of DRs, diabetic rats with contrast-induced nephropathy (CIN + DRs), melatonin-treated diabetic rats (MTDRs), and melatonin-treated CIN + diabetic rats (MTCIN + DRs). All groups except the HRs received 50 mg/kg/day streptozotocin (STZ) (Sigma, St. Louis, USA) within 0.1 M sodium citrate solution (pH: 4.50). Daily blood sugar measurements were performed through tail-end puncture using a Glucomax Ultra TD 4227 (Germany) blood sugar measurement device. In all STZ-administered groups, the blood sugar level was >400 mg/dL at the end of the 5th day. DRs did not receive any other treatment, whereas CIN + DRs and MTCIN + DRs were given 1.5 mg/kg of intravenous radiocontrast dye (Omnipaque, Amersham, Ireland) on the 35th day. In addition, MTDRs and MTCIN + DRs were given 20 mg/kg/day of intraperitoneal injection of MT (Sigma, St. Louis, USA) starting on the 28th day for seven days as described previously by Gazi et al. [10]. The MT solution was prepared within 2% ethanol solution diluted with water. All rats were sacrificed under thiopental sodium (5 mg/kg) anesthesia on the 36th day using blood evacuation via cardiac puncture and cervical dislocation. Kidney tissue samples were collected for histopathological and chemical analyses.

### 2.1. Histological Procedures for Light Microscopy and Electron Microscopy

Each kidney was fixed in 10% formalin solution for 48–55 h, dehydrated in a graded alcohol series, embedded in paraffin wax, and serially sectioned using a microtome (Leica RM2125RT). For light microscope histological examination, thin sections of 5 *μ*m thickness were taken from the same paraffin blocks. Slides were covered, and photographs were taken using a light microscope with a camera attachment (Nikon Eclipse E600, Japan).

For electron microscopy, kidneys were fixed in buffered 3% glutaraldehyde in 0.1 M phosphate solution, postfixed in 1% osmium tetroxide, dehydrated in a graded acetone series, and transferred to propylene oxide. After dehydration, specimens were embedded in Araldite CY 212. Sections were cut, using an ultratome (Nova LKB Bromma, Sweden), into 70–80 nm thin sections for histological evaluation at the ultrastructural level. After staining in 2% uranyl acetate and 0.4% lead citrate, sections were examined using a transmission electron microscope (Jeol 100 SX, Tokyo, Japan). Also, semithin sections (1 *μ*m) obtained with an ultramicrotome from the same araldite blocks were stained with toluidine blue and histopathologically examined using a light microscope.

### 2.2. Histopathological and Ultrastructural Examination

The kidneys were examined by light microscopy for histological evaluation of the following parameters: H&E and toluidine staining for inflammatory cell infiltrates, Armanni-Ebstein lesions, apoptotic cell death, glomerular changes and ultrastructural evaluation by electron microscopy for changes in glomerular basement membrane, podocytes and their pedicels, capillary structure, and mesangial cells.

### 2.3. Blood Samples Preparation and Analyses

Blood-containing tubes (Vacutainer SST, Becton Dickinson, USA) were centrifuged for 7 min at 3,000 rpm. Serum samples were placed in 2 mL Eppendorf tubes (Eppendorf, Germany) and stored at −80°C until the time of analysis. IL-6 (eBioscience, BMS625, USA), IL-33 (Cusabio, CSB-E14077r, China), and IL-1*β* (eBioscience, BMS630, USA) levels were measured using ELISA kits appropriate to the species in both kidney tissue and serum samples. Serum blood-urea nitrogen, creatinine, glucose, sodium, potassium, and calcium levels were measured in an autoanalyzer (AU 5800, Beckman Coulter, Japan) using original kits.

### 2.4. Lipid Peroxidation Determination

The level of lipid peroxidation (LPO) in kidney tissues was determined by estimating malondialdehyde (MDA) using the thiobarbituric acid test [11]. The kidney tissues were dissected, weighed, and homogenized in 10 mL of 100 g/L KCl. The homogenate (0.5 mL) was added to a solution containing 0.2 mL of 80 g/L sodium lauryl sulphate, 1.5 mL of 200 g/L acetic acid, 1.5 mL of 8 g/L 2-thiobarbiturate, and 0.3 mL of distilled water. The mixture was incubated at 98°C for 1 h. Upon cooling, 5 mL of n-butanol : pyridine (15 : 1) was added. The mixture was vortexed for 1 min and centrifuged at 4,000 g for 30 min. Subsequently, the absorbance of the supernatant was measured at 532 nm. The standard curve was obtained by using 1,1,3,3-tetramethoxypropane. The recovery rate was over 99%. The results were expressed as nmol MDA/g of tissue.

### 2.5. Myeloperoxidase Activity

Myeloperoxidase (MPO) activity was measured according to the modified method of Bradley et al. [12]. The homogenized samples were frozen and thawed three times and then centrifuged at 1,500 g for 10 min at 4°C. MPO activity in the supernatant was determined by adding 100 mL of the supernatant to 1.9 mL of 10 mmol/L phosphate buffer (pH 6.0) and 1 mL of 1.5 mmol/L o-dianisidine hydrochloride containing 0.0005% (wt/vol) hydrogen peroxide. The changes in absorbance at 450 nm of each sample were recorded on a UV-vis spectrophotometer. MPO activity in tissues was expressed as micromoles per min per milligram of tissue (*μ*mol/min/mg tissue).

### 2.6. Superoxide Dismutase Activity

Superoxide dismutase (SOD) activity was measured according to Sun et al. [13]. SOD estimation was based on the generation of superoxide radicals produced by xanthine and xanthine oxidase, which react with nitroblue tetrazolium (NBT) to form formazan. SOD activity was then measured at 560 nm by the degree of inhibition of this reaction and was expressed as mmol/min/mg of tissue.

### 2.7. Catalase Activity

Decomposition of H_2_O_2_ in the presence of catalase (CAT) was measured at 240 nm according to Aebi [14]. The CAT activity was defined as the amount of enzymes required to decompose 1 mmol of H_2_O_2_ per min at 25°C at pH 7.8. Results were expressed as mmol/min/mg of tissue.

### 2.8. Total Glutathione Levels

The amount of total glutathione (GSH) in kidney tissues was measured according to the method described by Sedlak and Lindsay [15], with some modifications. The kidney tissues were homogenized in 2 mL of 50 mMTris-HCl buffer containing 20 mM EDTA at pH 7.5. After adding 2 mL ethanol (to precipitate the proteins), the homogenate was centrifuged at 3,000 g for 40 min at 4°C. The supernatant was used to determine the GSH level using 5,5′-dithiobis(2-nitrobenzoic acid) (DTNB). The absorbance was measured at 412 nm. Subsequently, the GSH level of the kidney was expressed as nmol/g of tissue.

### 2.9. Data Analysis

All values were given as mean ± standard deviation. Nonnormally distributed data were analyzed by the nonparametric unpaired Mann-Whitney test. Multiple group comparisons were performed using ANOVA. A *p* value <0.05 was considered significant. All analyses were performed with the Statistica software program version 7.0 (StatSoft, Tulsa, OK, USA).

## 3. Results

In the present study, an LPO level which is accepted as an indicator of tissue damage as well as OxS was studied. In addition, the activities of SOD, CAT, and myeloperoxidase (MPX) enzymes and GSH levels were also measured to understand how the defense mechanisms were affected in kidney tissues.

### 3.1. Lipid Peroxidation Levels of Diabetic and Healthy Rats

**Figure 1:**
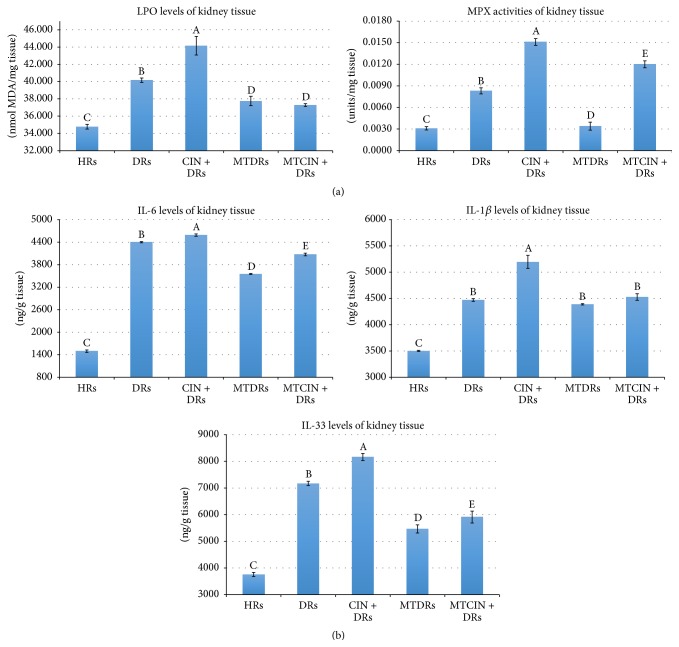
(a) The oxidative stress markers of groups. A: *p* < 0.05 for CIN + DRs versus HRs, and DRs regarding LPO and MPX levels. B: *p* < 0.05 for DRs versus HRs, regarding LPO and MPX levels. C: *p* < 0.05 for HRs versus MTDRs and MTCIN + DRs, regarding LPO and MPX levels. D: *p* > 0.05 for MTDRs versus MTCIN + DRs regarding LPO and MPX levels. E: *p* < 0.05 for MTCIN + DRs versus MTDRs regarding MPX levels. (b) The kidney tissue levels of IL-33, IL-6, and IL-1*β* of groups. A: *p* < 0.05 for CIN + DRs versus HRs, and DRs regarding IL-1*β* and IL-33 levels. B: *p* < 0.05 for DRs versus HRs, regarding IL-1*β*, IL-6, and IL-33 levels. C: *p* < 0.05 for HRs versus MTDRs and MTCIN + DRs, regarding IL-1*β*, IL-6, and IL-33 levels. D: *p* < 0.05 for MTDRs versus MTCIN + DRs regarding IL-6 and IL-33 levels. E: *p* < 0.05 for MTCIN + DRs versus CIN + DRs regarding IL-6 and IL-33 levels.

Kidney LPO levels of DRs were significantly increased when compared with HRs (40.1 ± 0.6 versus 34.7 ± 0.6, *p* < 0.05, resp.) (Figure 1(a)). When contrast dye was administered to DRs, kidney LPO levels were further increased when compared with HRs and DRs (44.1 ± 2.6 versus 40.1 ± 0.6 and 34.7 ± 0.6, *p* < 0.05 for both, resp.) (Figure 1(a)). In the present study, the kidney LPO levels were significantly decreased in both MTDRs and MTCIN + DRs when compared to DRs without MT treatment (37.7 ± 1.3 and 37.2 ± 0.3 versus 40.1 ± 0.6, *p* < 0.05, resp.) (Figure 1(a)). However, there was no statistically significant difference between MTDRs and MTCIN + DRs (37.7 ± 1.3 versus 37.2 ± 0.3, *p* > 0.05, resp.) (Figure 1(a)).

### 3.2. Myeloperoxidase Activity of Diabetic and Healthy Rats

Generally, MPX activity is used for showing the biochemical neutrophil activity of tissue. MPX uses hydrogen peroxide and chloride ions for production of hypochlorous acid that is a defense of neutrophil against bacteria and pathogens. In this study, the kidney tissue MPX activity was heightened in DRs and CIN + DRs when compared with HRs (0.0080 ± 001 and 0.015 ± 0.001 versus 0.003 ± 0.0006, *p* < 0.05) (Figure 1(a)). MPX levels of MTDRs and MTCIN + DRs were markedly decreased when compared with DRs and CIN + DRs (0.0034 ± 0.0014 and 0.012 ± 0.001 versus 0.0083 ± 0.001 and 0.015 ± 0.001, *p* < 0.05 for both, resp.) (Figure 1(a)).

### 3.3. Superoxide Dismutase Enzyme Activity of Diabetic and Healthy Rats

The SOD enzyme forms hydrogen peroxide by using superoxide radicals for elimination of these harmful radicals. Formed hydrogen peroxide is converted to water by the GSH peroxidase enzyme, which uses the GSH as a substrate. The increments or decrements of SOD activity were found to be closely related to the intensity of superoxide radicals. In the present study, the kidney levels of SOD activity were significantly raised in both DRs and CIN + DRs when compared with HRs (1.35 ± 0.02 and 1.44 ± 0.02 versus 1.28 ± 0.01, *p* < 0.05, resp.).

### 3.4. Glutathione Levels of Diabetic and Healthy Rats

Kidney levels of GSH were significantly diminished in DRs and CIN + DRs when compared with HRs (0.81 ± 0.01 and 0.78 ± 0.06 versus 097 ± 0.04, *p* < 0.05, resp.). GSH levels of MTDRs and MTCIN + DRs were markedly increased when compared with DRs and CIN + DRs (0.89 ± 0.01 and 0.85 ± 0.03 versus 0.81 ± 0.01 and 0.78 ± 0.06, *p* < 0.05 for both, resp.).

### 3.5. Catalase Levels of Diabetic and Healthy Rats

CAT transforms hydrogen peroxide to water by an enzymatic reaction. Kidney tissue CAT activity was increased in DRs and CIN + DRs when compared with HRs (0.008 ± 0.001 and 0.015 ± 0.001 versus 0.003 ± 0.0006, *p* < 0.05 for both, resp.). However, CAT activities of MTDRs and MTCIN + DRs significantly decreased compared with DRs and CIN + DRs (81.7 ± 3.6 and 84.6 ± 3.8 versus 90.9 ± 3.7 and 94.2 ± 3.3, *p* < 0.05 for all, resp.).

### 3.6. Kidney Tissue Levels of Interleukins of Diabetic and Healthy Rats

Kidney tissue IL-33 levels as well as IL-6 and IL-1*β* levels were significantly increased in DRs and CIN + DRs when compared with HRs (Table 1, Figure 1(b)) (*p* < 0.05, for all). IL-6 and IL-33 levels were found to be diminished in MTDRs and MTCIN + DRs when compared with DRs and CIN + DRs (*p* < 0.05, for all) (Table 1, Figure 1(b)).

### 3.7. Serum Interleukin Levels of Diabetic and Healthy Rats

In accordance with kidney tissue levels of ILs, serum levels of IL-33, IL-6, and IL-1*β* were found to be increased in DRs; moreover, contrast dye markedly boosted this rise (Table 1) (*p* < 0.05, for all). MT treatment significantly decreased serum IL-33 levels in both DRs and CIN + DRs (*p* < 0.05, for all) (Table 1).

### 3.8. Serum Creatinine Levels of Diabetic and Healthy Rats

Serum creatinine (SCr) levels were observed to increase significantly in DRs and CIN + DRs compared to HRs (0.35 ± 0.05 and 0.43 ± 0.05 versus 0.20 ± 0.06, resp., *p* < 0.05, for both). MT treatment significantly diminished SCr in both DRs and CIN + DRs (0.33 ± 0.05 and 0.37 ± 0.05, resp., *p* < 0.05, for both).

### 3.9. Histopathological Features of Healthy Rats, Diabetic Rats with Contrast-Induced Nephropathy, and Melatonin-Treated Diabetic Rats with Contrast-Induced Nephropathy

**Figure 2:**
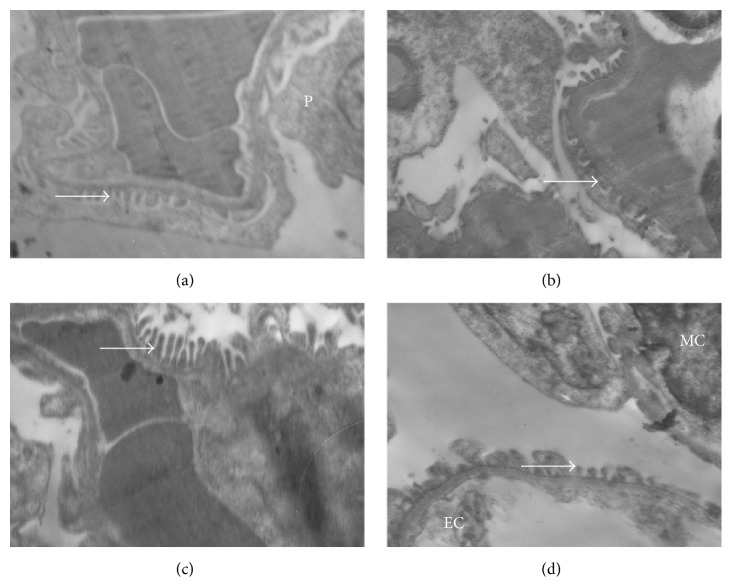
Electron microscopy findings of renal tissue samples of groups (×10000). Normal pedicels (white arrow) in the periendothelial area of renal tissue of HRs (Figure 2(a)). Closure of the filtration slits with atypic pedicels in the periendothelial area and electron-dense endothelial cytoplasm of CIN + DRs (Figure 2(b)). Basement membranes and pedicels of glomerulus of MTDRs (Figure 2(c)) and MTCIN + DRs (Figure 2(d)). P: podocyte; Ec: endothelial cell; MC: mesangial cell; MTDRs: melatonin-treated diabetic rats; MTCIN + DRs: melatonin-treated diabetic rats with contrast-induced nephropathy; DRs: diabetic rats; HRs: healthy rats; CIN + DRs: diabetic rats with contrast-induced nephropathy.

**Figure 3:**
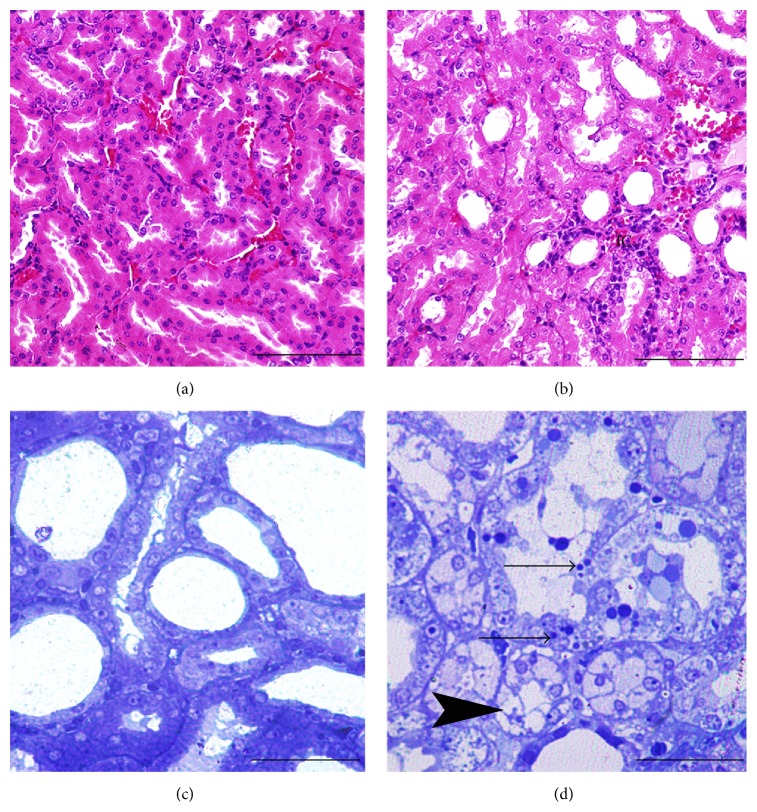
Morphology of renal tubular cells of HRs ((a), (c)) and CIN + DRs ((b), (d)). Histopathological changes such as mononuclear and polymorphonuclear leukocytes infiltration of interstitial cells (IC) of CIN + DRs (Figure 3(b)). Apoptotic tubular cells with membrane-bound apoptotic bodies (black arrow) and quite noticeable glucogenic vacuolization (arrow head) in tubular cells of CIN + DRs (Figure 3(d)). Note: histological micrographs were stained with H&E and had scale bars with 500 *μ* (Figures 3(a) and 3(b)). Histological micrographs were stained with toluidine blue and had scale bars with 150 *μ* (Figures 3(c) and 3(d)). HRs: healthy rats; CIN + DRs: diabetic rats with contrast-induced nephropathy.

In the ultrastructural and histopathological sections of kidney tissues, mesangial cells and capillaries of the glomerulus, podocytes (visceral epithelial cells) and their pedicels in Bowman's capsule, and cortical and medullary tubules appeared normal (Figures 2 and 3).

Renal tissues of the DRs showed histopathological changes as demolition in some glomeruli, inflammatory cell infiltration, transparent tubules that show glucogenic vacuolization (Armanni-Ebstein lesions), apoptotic cell death of renal tubules (Figures 2 and 3) and ultrastructural changes as closure of the filtration slits with atypic pedicels and basement membrane thickening (Figures 2(a)–2(d)), and mesangial hypertrophy in some glomeruli. In addition, electron-dense vesicle accumulation within endothelial cells was also observed (Figure 2(b)).

**Figure 4:**
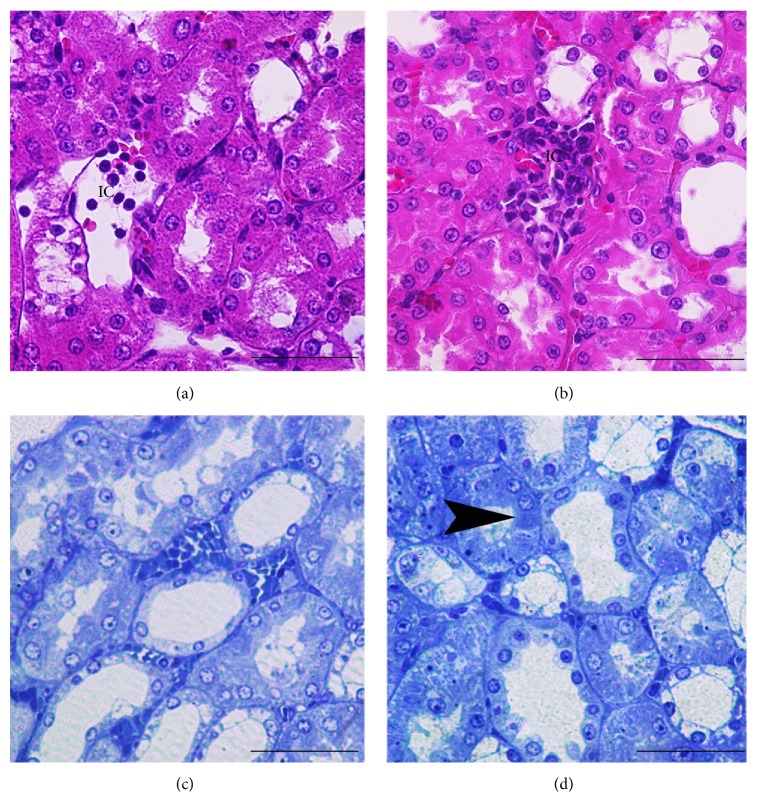
Morphology of renal tubular cells of MTDRs ((a), (c)) and MTCIN + DRs ((b), (d)). MTDRs showed similar patterns to HRs; however, inflammatory cell infiltration and vacuolization are demonstrated in MTDRs (arrow head). Histological section of the renal tissues contrast dye application after melatonin treatment (Figures 4(b) and 4(d)) showed mild interstitial leukocytes infiltration (Figure 4(b)) and Armanni-Ebstein lesions in tubule cells (arrow head) (Figure 4(d)). ((a) and (b)) Histological micrographs were stained with H&E and had scale bars with 150-micron size. Histological micrographs were stained with toluidine blue and had scale bars with 150-micron size (Figures 4(c) and 4(d)). MTDRs: melatonin-treated diabetic rats; MTCIN + DRs: melatonin-treated diabetic rats with contrast-induced nephropathy; HRs: healthy rats; CIN + DRs: diabetic rats with contrast-induced nephropathy.

These histopathological changes of the MTDRs were less when compared with the DRs. MTDRs also had regenerative changes (Figures 2 and 4). Apoptosis, necrotic changes, and Armanni-Ebstein lesions were found to be diminished in MTDRs (Figures 4(a)–4(d)). Also, basement membranes, pedicel, and endothelial cells generally appeared normal (Figures 2(a)–2(d)). However, local interstitial inflammatory cells were observed in MTDRs, although they were found to be less than DRs (Figure 4). In contrast, we observed less regenerative changes in MTCIN + DRs (Figures 2 and 4).

## 4. Discussion

There are three main findings of the present study. First, kidney tissue and serum levels of IL-33 as well as other inflammatory cytokines and OxS markers were found to be significantly increased in DRs, and further increase was also determined in contrast-applied DRs. Second, both kidney tissue and serum levels of OxS markers, inflammatory cytokines, and IL-33 were found to be significantly diminished in MTCIN + DRs compared with the groups without MT treatment. Third, these findings are also supported by the histopathological findings determined by electron and light microscope. This study is the first to demonstrate the possible role of IL-33 in the pathogenesis of DRs with CIN and the preventive effect of MT in this situation.

Increased serum glucose levels along with chronic low grade inflammation, OxS, advanced glycation end products (AGE), sorbitol accumulation, hexosamine, and protein kinase C (PKC) pathway activation are well-known features of diabetes mellitus [7]. One of the most important consequences of these alterations is DKD. Diabetic patients might need further investigations with contrast using methods such as computed tomography imaging for clarifying the situations that are commonly faced. The exact mechanism of CIN is also not fully elucidated. Contrast agents have direct toxic effects on renal tubular cells and renal hemodynamics, and based on previous studies, oxygen radicals might play a major role as the primary physiological insult [16, 17]. Infusion of contrast agents increases in osmotic load and viscosity, and thus hypoxemia of the renal medulla and renal free radical production through postischemic OxS occur [17]. In the present study, we found that markers of OxS are increased and antioxidants are decreased in DRs and DRs with CIN, in accordance with the current literature [7, 18, 19].

Previous clinical studies have established an association between a rise in inflammatory markers, including IL-6, IL-18, and TNF-*α*, and DKD [20]. Among several cytokines that may be relevant to the development of DKD, it has been demonstrated that serum IL-18 levels are markedly elevated in patients with type 2 diabetes with microalbuminuria when compared to those with normoalbuminuria, while IL-6 rises at a later time, when clinical macroalbuminuria is present [21].

According to a hypothesis adopted by many authors, OxS might be the main trigger system for the initiation and progression of DKD [7]. Another novel concept is that chronic inflammation may result in local disruption of insulin signaling, leading to the initial development of both micro- and macrovascular complications of diabetes [22]. However, the exact trigger of the chronic complications of diabetes remains mysterious. Hence, further experimental data are needed to characterize the causative role of cytokines in the development of DKD. In this regard, we hypothesized that IL-33, also named alarmin, might be a candidate as a trigger cytokine in DRs with CIN. IL-33 is a proinflammatory cytokine that can be released from necrotic cells, binds the ST2R on immune cells, and increases secretion of cytokines, with resultant inflammation [23, 24]. The exact role of ST2/IL-33 axis remains mysterious in diabetes. For instance, BALB/c mice were found to be resistant to the induction of T cell-mediated diabetes when multiple low doses of STZ were administered [25]. Additionally, BALB/c mice with the ST2 gene deleted were also found to be more prone to STZ-induced diabetes [26]. Recently, Karatas et al. [27] showed that IL-33 expression was typically located in the peritubular and intraglomerular capillaries. They also found 6-fold (diabetic mice at the 4th month versus healthy mice, *p* < 0.01) and 5.8-fold (diabetic mice at 6th month versus healthy mice, *p* < 0.01) increases in IL-33 protein expression in western blots of whole kidney tissue, and assessment of IL-33 with ELISA confirmed their results (IL-33 levels 0.409 pg/*μ*g diabetic mice at the 6th month versus 0.164 pg/*μ*g healthy mice, *p* < 0.01). These results also support our hypothesis that IL-33 may participate in the initiation and progression of DKD.

The exact function of IL-33 in the nucleus has not been established; however, IL-33 can activate the transcription factor AP-1 as well as nuclear factor-kappa B (NF-*κ*B) [28]. Hence, IL-33 has intracellular transcriptional regulatory properties as well as a proinflammatory cytokine. Recently, Akcay et al. [8] clearly demonstrated that IL-33 plays a central role in the pathogenesis of cisplatin-induced acute kidney injury via invoking CD4^+^ T lymphocyte to the kidney. The major proinflammatory effect of IL-33 is to induce CD4^+^ Th2 cell responses via the ST2R [29]. The present study is also unique in that it shows an effect of a nonimmune stimulus, contrast dye, on IL-33. In this regard, our study demonstrates that IL-33 is expressed in the kidneys of both DRs and contrast-applied DRs.

Tubulointerstitial fibrosis is one of the most important features in the progression of DKD. Inflammatory mediators, including cytokines, have important roles during the recruitment of inflammatory cells; these drive the fibrogenic process. Interleukins including IL-1*β* and IL-33 were found be very active during the fibrogenic process [30]. Recent studies also demonstrated that inhibition of inflammatory cell infiltration might contribute to the control of renal tubulointerstitial fibrosis. For instance, melatonin was found to be beneficial in terms of leukocyte infiltration, via reduction in the expression of NF-*κ*B in a rat model of unilateral ureteral obstruction [31]. In another rat model of carbon tetrachloride nephrotoxicity, interstitial mononuclear cell infiltration and fibrosis of kidney were ameliorated by melatonin treatment [32]. In this context, according to the present study results, it is wise to consider that melatonin treatment might reduce tubulointerstitial fibrosis via IL-33 related pathways.

Previous studies have reported that MT can prevent drug-induced nephropathy including STZ and contrast dye [10, 18]. The administration of MT results in a rapid rise in blood MT concentrations. Since MT has both highly lipophilic and hydrophilic properties, it passes rapidly through all biological membranes and enters the cells and their subcellular compartments when administered intraperitoneally or intravenously. The widespread subcellular distribution of MT may allow it to reduce oxidative damage in both the lipid and aqueous environments of the cell. This is an advantage of MT over some other antioxidants, which penetrate cells more slowly.

However, there has been no data regarding whether MT is preventive against both DKD and CIN situations together. In our daily practice, the physicians use diagnostic tools such as computed tomography with contrast dye or do angiography when needed in patients with DKD. The risk of morbidity and mortality was found to be higher in patients with DKD when exposed to contrast dye [33]. Our study is unique in that it has demonstrated that pretreatment with MT significantly improved CIN in patients with DKD. Structural features of MT, including lipophilicity, hydrophilicity, and electron-richness, provide additional beneficial effects of this molecule in terms of antioxidant capacity and insulin secretion effect via the MT1 receptor [19]. MT activates several antioxidative enzymes, including GSH peroxidase, SOD, and lipid peroxidase [34]. MT also easily crosses cellular membranes and provides on-site protection against free radical-mediated damage to biomolecules. Although the exact mechanism of CIN is not very well known, deleterious factors such as vasoconstriction, tubular obstruction, mitochondrial injury, and plasma membrane toxicity have been implicated. Hence, MT might be a promising agent to protect renal functions in DKD and CIN via its antioxidant and antihyperglycemic effect.

Between 1990 and 2000, many studies have demonstrated the possible interaction between MT and the immune system [35, 36]. MT's immunoenhancing effect depends upon its ability to enhance the production of cytokines as well as its antiapoptotic and antioxidant action. MT has been proposed to regulate the immune system by affecting cytokine release from immunocompetent cells [37]. Additionally, MT was found to augment CD4+ lymphocytes and decrease CD8+ lymphocytes in rat submaxillary lymph nodes [38]. MT enhances the production of IL2, IFN-*γ*, and IL-6 by cultured human mononuclear cells [39]. MT might also increase the production of IL-1, IL-6, and IL-12 via activating monocytes [40]. Repetitive stimulation of T helper (Th) cells via MT might force Th cells to switch to Th1 lymphocytes that enhance the release of IL-2 and IFN-*γ*. These latter cytokines can particularly invite macrophages and other phagocytes to the inflammation site. In addition, MT supplementation was found to increase CD4^+^ Th production [35]. Besides the release of proinflammatory Th-1 cytokines, Raghavendra et al. [41] also demonstrated that MT might activate an anti-inflammatory Th-2-like immune response. Collectively, these data indicate that MT possesses important immunoenhancing properties and suggest that MT may favor both Th-1 and Th-2 responses. IL-33 stimulation was found to be crucial for the transformation of CD4 T cells into the Th-2 subgroup [9]. We did not measure the levels of CD4+ lymphocytes before and after MT treatment. However, the beneficial effect that we show in the MTDRs and MTCIN + DRs might depend on a CD4 + lymphocytes-IL-33-MT circle.

In summary, contrast dye application induces an increase of IL-33 in the kidneys in DKD. Inhibition of IL-33 with MT provides functional and histologic protection against CIN, demonstrating that IL-33 might be a candidate for mediating CIN in DKD. Inhibition of IL-33 and oxidants with MT pretreatment offers therapeutic potential in contrast dye-applied diabetic patients.

## Figures and Tables

**Table 1:** Serum and kidney tissue levels of interleukins among groups.

Parameters	HRs (*n* : 6)	DRs (*n* : 6)	CIN + DRs (*n* : 6)	MTDRs (*n* : 6)	MTCIN + DRs (*n* : 6)
Kidney tissue IL-33 (ng/g tissue)	3756 ± 184	7171 ± 194^¶^	8163 ± 325^*∗*^	5463 ± 373^¥^	5910 ± 552^#^
Kidney tissue IL-6 (ng/g tissue)	1495 ± 81	4400 ± 41^¶^	4589 ± 77^*∗*^	3552 ± 33^¥^	4072 ± 86^#^
Kidney tissue IL-1*β* (ng/g tissue)	3501 ± 26	4470 ± 64^¶^	5194 ± 304^*∗*^	4387 ± 32	4527 ± 151
Serum IL-33 (ng/dL)	101 ± 4.7	144 ± 2.1^¶^	150 ± 4.1^*∗*^	115 ± 2.3^¥^	122 ± 2.8^#^
Serum IL-6 (ng/dL)	106 ± 11.3	149 ± 7.9^¶^	226 ± 7.5^*∗*^	101 ± 4.4^¥^	109 ± 6.5
Serum IL-1*β* (ng/dL)	27.9 ± 1.2	33.8 ± 3.3^¶^	46.1 ± 2.7^*∗*^	35.8 ± 6.0	32.7 ± 0.4
Serum creatinine (ng/dL)	20 ± 6	27 ± 4^¶^	43 ± 5^*∗*^	22 ± 3^¥^	33 ± 5^#^

HRs: healthy rats, DRs: diabetic rats, CIN + DRs: diabetic rats with contrast-induced nephropathy, MTDRs: melatonin-treated diabetic rats, and MTCIN + DRs: melatonin-treated diabetic rats with contrast-induced nephropathy.

¶: diabetic rats versus healthy rats, *p* < 0.05.

*∗*: diabetic rats with CIN versus healthy rats, *p* < 0.05.

¥: melatonin-treated diabetic rats versus diabetic rats, *p* < 0.05.

#: melatonin-treated diabetic rats with CIN versus diabetic rats with CIN, *p* < 0.05.
